# Effect of Karas (*Aquilaria malaccensis*) on Male Reproductive Organs and Sperm Quality in Adult Sprague Dawley Rats

**DOI:** 10.21315/tlsr2023.34.1.13

**Published:** 2023-03-31

**Authors:** Norahidah Zaidi, Mohd Nizam Haron, Connie Fay Komilus, Fathurrahman Lananan, Ha Hou Chew, Nadzifah Yaakub, Asmad Kari

**Affiliations:** School of Animal Science, Aquatic Science and Environment, Faculty of Bioresources and Food Industry, Universiti Sultan Zainal Abidin (UniSZA), 22200 Besut, Terengganu, Malaysia

**Keywords:** Karas, *Aquilaria malaccensis*, Organ Reproduksi Lelaki, Kualiti Sperma, Karas, *Aquilaria malaccensis*, Male Reproductive Organ, Sperm Quality

## Abstract

Reproductive health and male fertility are closely related to dietary practices. In recent years, Malaysia has shown a lot of interest in using herbal plants as dietary supplements or in the treatment of numerous diseases. *Aquilaria malaccensis*, commonly known as karas or gaharu, has recently gained attention for its potential to cure many diseases due to its pharmacological properties. However, studies on its effect on male fertility and reproductive organs are very scarce. This study was conducted to determine the effect of *A. malaccensis* on male reproductive organs’ weight (testis, epididymis, prostate gland and seminal vesicle) and sperm quality (sperm count, sperm morphology and sperm motility) in adult Sprague Dawley rats. Twenty-four male Sprague Dawley rats were allocated into four treatment groups; Control (C: 1 mL of distilled water, *n* = 6), Treatment 1 (T1: 1 g *A. malaccensis*/kg body weight, *n* = 6), Treatment 2 (T2: 2 g *A. malaccensis*/kg body weight, *n* = 6) and Treatment 3 (T3: 3 g *A. malaccensis*/kg body weight, *n* = 6), respectively. Distilled water and *A. malaccensis* were administered by oral gavage once daily for 28 days. The rats were euthanised on Day 29 for assessment of reproductive organs’ weight and sperm quality. Result shows that weight of testis, epididymis, prostate gland, seminal vesicle and sperm motility did not differ (*p* > 0.05) among control and treated groups. A significant increase (*p* < 0.05) of sperm number (1.36 **×** 10^−6^) and a decrease (*p* < 0.05) in percentage of the abnormal sperm (8.17%) were observed in T1 group when compared to Control group. Incremental dosage of *A. malaccensis* seemed to decrease number of sperm (T3: 0.78 **×** 10^−6^ < T1: 1.36 **×** 10^−6^ with *p* < 0.05) and increase percentage of abnormal sperm (T3: 18.83% > T2: 12.17% > T1: 8.17% with *p* < 0.05). In conclusion, the administration of either 1, 2 or 3 grams of *A. malaccensis* did not alter the reproductive organs’ weight and sperm motility. However, the higher concentration of *A. malaccensis* consumed by the rats seemed to have detrimental effects on the number and morphology of sperm.

## Abstract (fr)

Kesihatan reproduktif dan kesuburan lelaki berkait rapat dengan amalan pemakanan. Beberapa tahun kebelakangan ini, Malaysia telah menunjukkan minat yang mendalam dalam penggunaan tumbuhan herba sebagai makanan tambahan atau rawatan dalam pelbagai penyakit. *Aquilaria malaccensis*, yang biasanya dikenali sebagai karas atau gaharu, telah mendapat perhatian kerana berpotensi dalam menyembuhkan pelbagai penyakit disebabkan sifat farmakologinya. Walau bagaimanapun, kajian terhadap kesannya kepada kesuburan lelaki dan organ reproduksi sangat terhad. Oleh itu, kajian ini dijalankan untuk menentukan kesan *A. malaccensis* terhadap berat organ reproduksi lelaki (testis, epididimis, kalenjar sperma dan vesikel mani) dan kualiti sperma (kepekatan sperma, morfologi sperma dan motiliti sperma) terhadap tikus dewasa Sprague Dawley. Dua puluh empat tikus jantan Sprague Dawley telah dibahagikan kepada empat kumpulan rawatan; Kawalan (C: 1 mL air suling, *n* = 6), Rawatan 1 (T1: 1 g *A. malaccensis*/kg berat badan, *n* = 6), Rawatan 2 (T2: 2 g *A. malaccensis*/kg berat badan, *n* = 6) dan Rawatan 3 (T3: 3 g *A. malaccensis*/kg berat badan, *n* = 6). Air suling dan *A. malaccensis* diberi secara suapan gavaj sekali sehari selama 28 hari. Semua tikus dimatikan pada hari ke-29 untuk penilaian terhadap berat organ reproduksi dan kualiti sperma. Hasil yang diperolehi menunjukkan bahawa berat testis, epididimis, kalenjar prostat, vesikel mani dan motiliti sperma tiada perbezaan (*p* > 0.05) antara kumpulan kawalan dan rawatan. Peningkatan yang signifikan (*p* < 0.05) dalam kepekatan sperma (1.36 × 10^−6^) dan penurunan yang signifikan (*p* < 0.05) dalam peratusan sperma tidak normal (8.17%) dapat dilihat dalam kumpulan T1 jika dibandingkan dengan kumpulan rawatan. Peningkatan dos *A. malaccensis* telah menunjukkan pengurangan kepekatan sperma (T3: 0.78 × 10^−6^ < T1: 1.36 × 10^−6^ dengan *p* < 0.05) dan peningkatan peratusan sperma tidak normal (T3: 18.83% > T2: 12.17% > T1: 8.17% dengan p < 0.05). Kesimpulannya, pemberian sama ada 1, 2 atau 3 gram A. malaccensis tidak mengubah berat organ pembiakan dan motiliti sperma. Walau bagaimanapun, kepekatan *A. malaccensis* yang lebih tinggi yang dimakan oleh tikus nampaknya mempunyai kesan buruk terhadap bilangan dan morfologi sperma.

**Highlights**
- All rats administrated with *Aquilaria malaccensis* leaves extract (1 g, 2 g and 3 g) did not show any effect on the weight of reproductive organs.
- Oral treatment of 1 g *Aquilaria malaccensis* leaves extract demonstrated increment in sperm number and decrement in percentage of abnormal sperm.
- Adverse effect was observed with higher dosage of *Aquilaria malaccensis* extract (2 g and 3 g) supplemented to the rats.

## INTRODUCTION

Reproductive health is vital for the survival of all living things. However, the global deterioration of male reproductive health and fertility is a major concern when Malaysia has also shown a declining trend in the total fertility rate over the past few decades (Dutta & Sengupta 2018; Durairajanayagam 2018; Cariati *et al.* 2019; Mann *et al.* 2020; Jegasothy *et al.* 2021). The reproductive system of males is crucially essential for producing male gametes and their transportation to the female reproductive tract for successful fertilisation (Mohanty & Singh 2017). Male fertility can be influenced by a variety of factors including physiological stress, heat stress, exposure to electromagnetic radiation, advanced paternal age and the use of illicit drugs (Semet *et al.* 2017; Durairajanayagam 2018; Ilacqua *et al.* 2018). According to Durairajanayagam (2018), dietary practices also might be one of the factors that contribute to men’s fertility reduction as some chemical constituents may impair male reproductive function by damaging sperm DNA. For example, excess of alcohol consumption may result in uncontrolled production of reactive oxygen species (ROS) and a rapid loss in antioxidant capacity (Agarwal *et al.* 2020). In addition, disturbance in the intricate balance between the production and scavenging of ROS is a common underlying mechanism associated with male infertility (Agarwal *et al.* 2006; Santos-Filho *et al.* 2007). This improper balance between ROS generation and scavenging activity leads to oxidative stress and damaging the sperm cells (Jimoh *et al.* 2021). This circumstance eventually disrupts spermatogenesis, sperm quality, steroidogenesis, and sexual functionality, leading to male infertility (Agarwal *et al.* 2006).

Recently, increasing interest in herbal medicines is proliferating especially in treating fertility problems in both males and females. This is because most people believe that these herbal medicines are safe to be consumed and harmless as they are from natural resource and have been used as traditional medicine for years (Priya *et al.* 2012; Sekar *et al.* 2014; Wan *et al.* 2017; Van & Prinsloo 2020; Pauzi *et al.* 2021). In addition, people from various cultures have used herbal plants as traditional medicine since ancient times to treat many types of diseases such as diarrhoea, diabetes and asthma. In recent years, a multilateral approach in using herbal plants as alternative therapies has emerged in many countries (Modaresi *et al.* 2008; Mohajeri *et al.* 2009). Herbal plants offer more advantages than other treatments because they are less invasive and affordable (Mohammadi *et al.* 2013; Azahari *et al.* 2015; Roy *et al.* 2018; Habibi *et al.* 2019; Salleh *et al.* 2021). However, most of the application of these herbal plants as dietary supplements or in treatment of numerous diseases are used without proper knowledge of their function or safe dosage to be consumed which might cause harmful effects to the users (Priya *et al.* 2012).

In Malaysia, there a few types of herbals that are commonly used to treat many kinds of diseases traditionally such as *Ficus deltoidea*, *Labisia pumila*, *Cosmos caudatus* and *Nigella sativa* (Afzan *et al.* 2019; Uzbek & Shahidan 2019; Mohamad *et al.* 2019; Zakaria *et al.* 2021). Among these herbals, Aquilaria species, locally known as Karas or Gaharu, is identified as one of the most valuable plants in Malaysia due to their high-value fragrant resinous wood (agarwood) (Liu *et al.* 2019; Desa *et al.* 2021; Rozihawati *et al.* 2022). It is classified under the family of Thymelaeaceae and widely distributed in south and south-east Asia such as Malaysia, India, Thailand and Indonesia. Around 25 species of Aquilaria have been reported, such as *A. malaccensis*, *A. crassna*, *A. sinensis*, *A. subintegra*, *A. microcarpa* and *A. hirta* (Zulkifle *et al.* 2018). In Malaysia, *A. malaccensis* is the most popular Aquilaria species that produce agarwood resins when the wood is being infected by pathogens or wounded (Azah *et al.* 2008; Ahmad 2016; Ramli *et al.* 2022). Historically, this plant has been used for medicinal, aromatic and religious purposes for thousands of years by many people (Kharnaior & Thomas 2021). In addition, this plant is commonly used in folk medicine to treat many diseases such as diabetes, arthritis, gout and asthma (Hashim *et al.* 2016; Adhikari *et al.* 2021; Indrisari *et al.* 2021). Recently, many studies have proven that various parts, including seeds, leaves, wood and roots, have potentials to be beneficial in the pharmaceutical field (Razak *et al.* 2019a; 2019b).

Studies have demonstrated some beneficial effects of Aquilaria species. The leaf extract of this plant was reported to exhibit potent antioxidant, anti-microbial, analgesic, antipyretic, anti-inflammatory, anti-hyperglycemic and anti-microbial activities (Kamonwannasit *et al.* 2013; Hendra *et al.* 2016; Razak *et al.* 2019a; 2019b). Several bioactive compounds such as flavonoids, alkaloids, tannins, saponins and phenolic compounds can be found in *A. malaccensis* leaves extract (Derouiche *et al.* 2019; Eissa *et al.* 2020; Batubara *et al.* 2021). Previous studies also have reported the presence of steroids, flavonoids, triterpenoids, tannins and alkaloids in methanol leaves extract of *A. malaccensis* (Nik *et al.* 2014). A phytochemical finding also revealed the presence of flavonoid glycosides, 2-(2-phenylethyl) chromenes, lignans and diterpenoids in this plant extract (Musa *et al.* 2019). Numerous studies have been performed to examine the possible use of the extracts in diabetes care, arthritis, infection, inflammation, oxidation stress and cancer (Zulkifle *et al.* 2018; Hegde *et al.* 2018; Mokhtar *et al.* 2021). Previous studies of agarwood also reported the capability of Aquilaria species to demonstrate laxative and antidepressant effects in humans (Kakino *et al.* 2010; Wang *et al.* 2018).

Although there were studies conducted on rats to determine the effects of *A. malaccensis* leaves aqueous extract (0.1 g/kg–1.0 g/kg) on male sexual behaviour, sperm fertilising capacity and ameliorative effects on male reproductive toxicity, there is still lack of information and insufficient scientific investigation on its effect on male fertility and reproductive function (Ismail *et al.* 2019; Musa *et al.* 2019; Razak *et al.* 2019a; 2019b). Therefore, this study was conducted to determine the effect of *A. malaccensis* leaves aqueous extract on weight of reproductive organs and sperm quality on adult male of Sprague Dawley rats. This study may provide information on effects of *A. malaccensis* leaves aqueous extract whether it has side effects onto male reproduction system, especially for those who consume it as a supplement and medicine.

## MATERIALS AND METHODS

### Plant Material

The leaves of *A. malaccensis* were collected from Merchang Forest Reserve, Terengganu State Forestry Department, Terengganu with assistance from Assistant Field Officers of the respected department in species identification and selection purposes. Identification and selection of leaves were done according to the Forestry Department of Peninsular Malaysia Guideline (Forestry Department of Peninsular Malaysia 2012). The age of the selected trees was in the range of 20 years–45 years. The leaves were recognised based on their elliptical-oblong shape and size (length; 6 cm–8 cm and width; 3 cm–3.5 cm). The leaves collection was done in the morning. During the process, several bunches of leaves with two different maturity stages (young and old) were collected.

### Leaf Extract Preparation

Fresh leaves of *A. malaccensis* were air-dried in an empty room (23**°**C–26°C) for three consecutive days. These dried leaves were then pulverised into powder form with a waring blender (Waring Commercial, United State of America). The leaves powder (g) was soaked in distilled water (mL) with a ratio of 1:15 and at room temperature for 24 h (Alipieva *et al.* 2010). After soaking, the mixture was placed in a sonicator (Wisd, Korea) for 20 min and filtered to remove the leaves powder. Filtrate was then centrifuged at 4000 rpm (25**°**C) for 20 min to collect the supernatant (Rashid *et al.* 2020). Extraction procedures were carried out three times and pooled filtrates were then concentrated at 40**°**C with a rotary vacuum evaporator (Heidolph, Germany) to separate the solvent from the crude extract (Samsulrizal *et al.* 2011). The total yield of 2 kg brown crude extract was kept in universal bottles and stored at 4**°**C until further use.

### Animal Preparation

Twenty-four (*n* = 24) adult male Sprague-Dawley rats aged 8 to 9 weeks, weighed between 250 g to 300 g were selected and acclimatised for a week before starting the treatment. Animals were housed in standard-sized cages (19 × 13.5 × 8 inches) with six rats per cage. They were caged at 20°C–24°C, maintained under standard laboratory conditions with 50 ± 10% humidity and a cycle of 12-h light and 12-h dark. Standard laboratory rat feed (Gold Coin Feed Mills Sdn Bhd, Malaysia) and water were given at *ad libitum*. The feed was specifically formulated to meet all of their nutritional requirements. All experiments were carried out in the Animal Treatment laboratory at the Faculty of Bioresources and Food Industry, Universiti Sultan Zainal Abidin (UniSZA). According to the Organisation for Economic Co-operation and Development (OECD) Test Guideline 407 (OECD 2008), all ethical themes of studies on animals were conducted. All experimental animal procedures were approved by the Universiti Sultan Zainal Abidin Animal and Plant Research Ethics Committee with reference number UAPREC/04/044.

### Experimental Design

Twenty-four male rats were randomly categorised into four groups (*n* = 6/group), consisting of Control and three different Treatment groups. Control group was administered with 1 mL of distilled water. Meanwhile, Treatment groups were administered with *A. malaccensis* extract at 1 g/kg (T1), 2 g/kg (T2) and 3 g/kg (T3) on body weight of rat. The plant extract was reconstituted in distilled water according to the doses required for each treatment group. They were given once daily in the morning for 28 days continuously by force-feeding with oral gavage. Individual body weights of rats in each group were recorded daily before administering distilled water and *A. malaccensis* extract. Food and water intake, colour of urine, and consistency of stool were monitored daily for a period of 28 days. All animals were sacrificed after full treatment by cervical dislocation for blood and organs collection (Shrestha *et al.* 2018).

### Reproductive Organ Weight

**Figure 1:**
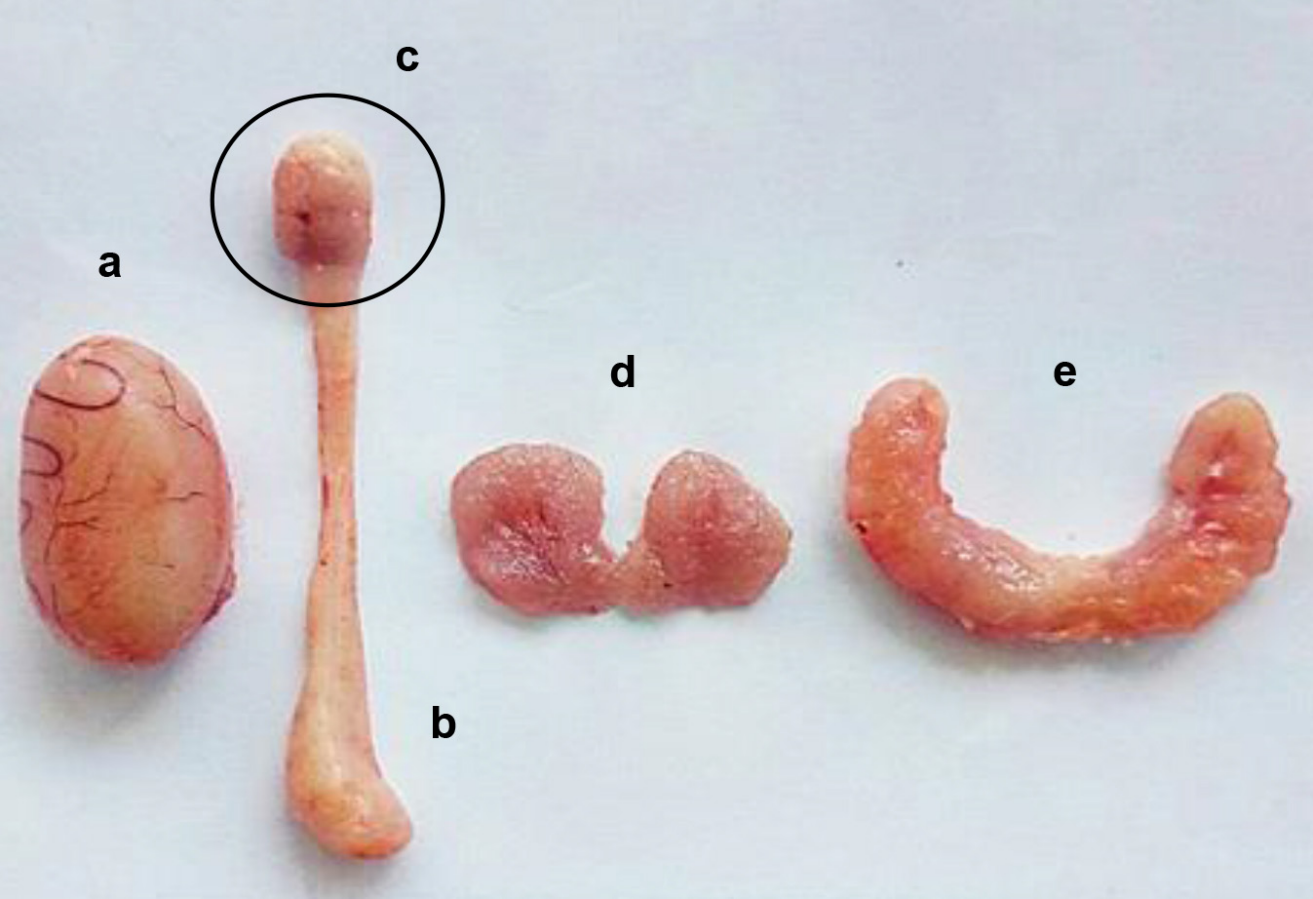
Male reproductive organs. (a) Testis; (b) Epididymis; (c) Cauda epididymis; (d) Prostate gland; (e) Seminal vesicle.

Reproductive organs, including the testis, epididymis, prostate gland, and seminal vesicle, were collected immediately after dissection (Fig. 1). Any attached tissues or visible fat at the organs were trimmed off completely. Samples were then washed thrice in normal saline and dried with filter paper folds to blot away any blood and fluid. The removed organs were then weighed by using an analytical balance (Radwag, Poland).

### Sperm Count

Cauda epididymis from left side was removed and minced with a sharp scissor in 2 mL of normal saline followed by filtration through 80 μm nylon mesh. Filtrate was then mixed with two drops of eosin Y and kept for 30 min. An aliquot of the stained epididymal suspension was then removed using a pipette (used for white blood cell counts) up to the 0.5 mark and then diluted with normal saline to the level marked 11 on the pipette. The dilution (10 μL) was then placed under the coverslip of each side of the Neubauer hemocytometer (Hawksley, United Kingdom). The Neubauer hemocytometer was then placed under a microscope and viewed at 400× magnifications. Counting was done manually in ruled squares located in Neubauer’s counting area. Numbers of sperm (with head and tail) in five squares in the centre grid of both sides were calculated and averaged. These methods were carried out and assessed according to the previous study (Narayana *et al.* 2002; Mohamed *et al.* 2012). As the sperm observation was done manually, the count has been repeated five times to minimise the counting error. Formula used for calculating sperm count was dilution factor × sperm counted × (0.05 × 10^6^) (Narayana *et al.* 2002; Haron *et al.* 2010).

### Sperm Morphology

For sperm morphology, the same epididymal suspension (10 μL) used for sperm count was placed on a clean glass slide and smeared over the slide. Slides were left to dry at room temperature for 24 h and then examined. A total of two hundred sperms from each animal were examined randomly under a light microscope at 400× magnifications (Haron *et al.* 2010; Zakaria & Haron 2020). As the sperm observation was done manually, the count has been repeated five times to minimise the counting error. Number of abnormal sperms (head, mid-piece or tail) were then calculated and expressed as a percentage.

### Sperm Motility

The observation of sperm motility was performed immediately after sperm collection. Vas deferens was taken out after sacrifice and placed in 1 mL of normal saline at room temperature. The content of vas deferens was then squeezed out. The suspension (3 μL) was placed on Makler’s counting chamber for observation under a microscope at 100**×** magnifications (Makler 1980). Sperm motility was measured based on their speed and movement condition (graded 0 to 3) (Walczak *et al.* 2013). A score of 0 (0% motile) was categorised as no motility, while a score of 3 (>70% motile) was categorised as maximum perceived motility (where the sperm became a vigorously swirling cell mass). Intermediate scores (1 and 2) were located between these two extremes, where the score of 1 (<30% motile) representing non-progressive and 2 (30%–70% motile) representing slow-progressive. The measurement was then expressed as a percentage.

### Statistical Analysis

Statistical analysis was carried out by using Statistical Package for Social Science (SPSS) 21.0 software. Distribution and data variance were analysed using whisker-box plot and Levene’s test, respectively. Numerical data with normal distribution and homogenous variance were analysed using one-way ANOVA parametric test followed by Tukey’s post hoc test. The Chi-square test for independence data was used to analyse the categorical data. Value of *p* < 0.05 was considered to be statistically significant.

## RESULTS

### Weight of Reproductive Organs

Table 1 shows the results for the weight of reproductive organs, including testis, epididymis, prostate gland, and seminal vesicle. No significant difference (*p* > 0.05) was seen in the mean weight of the reproductive organs among all groups.

### Sperm Count

Table 2 shows the results for sperm concentration and sperm morphology. Data for sperm concentration was normally distributed and one-way ANOVA test indicated that there were significant differences (*p* < 0.05) in the mean of sperm concentration among groups. The T1 group was seemed to have the highest sperm concentration compared to control and other groups. However, the concentration of sperm decreased with incremental dosage.

### Sperm Morphology

**Figure 2:**
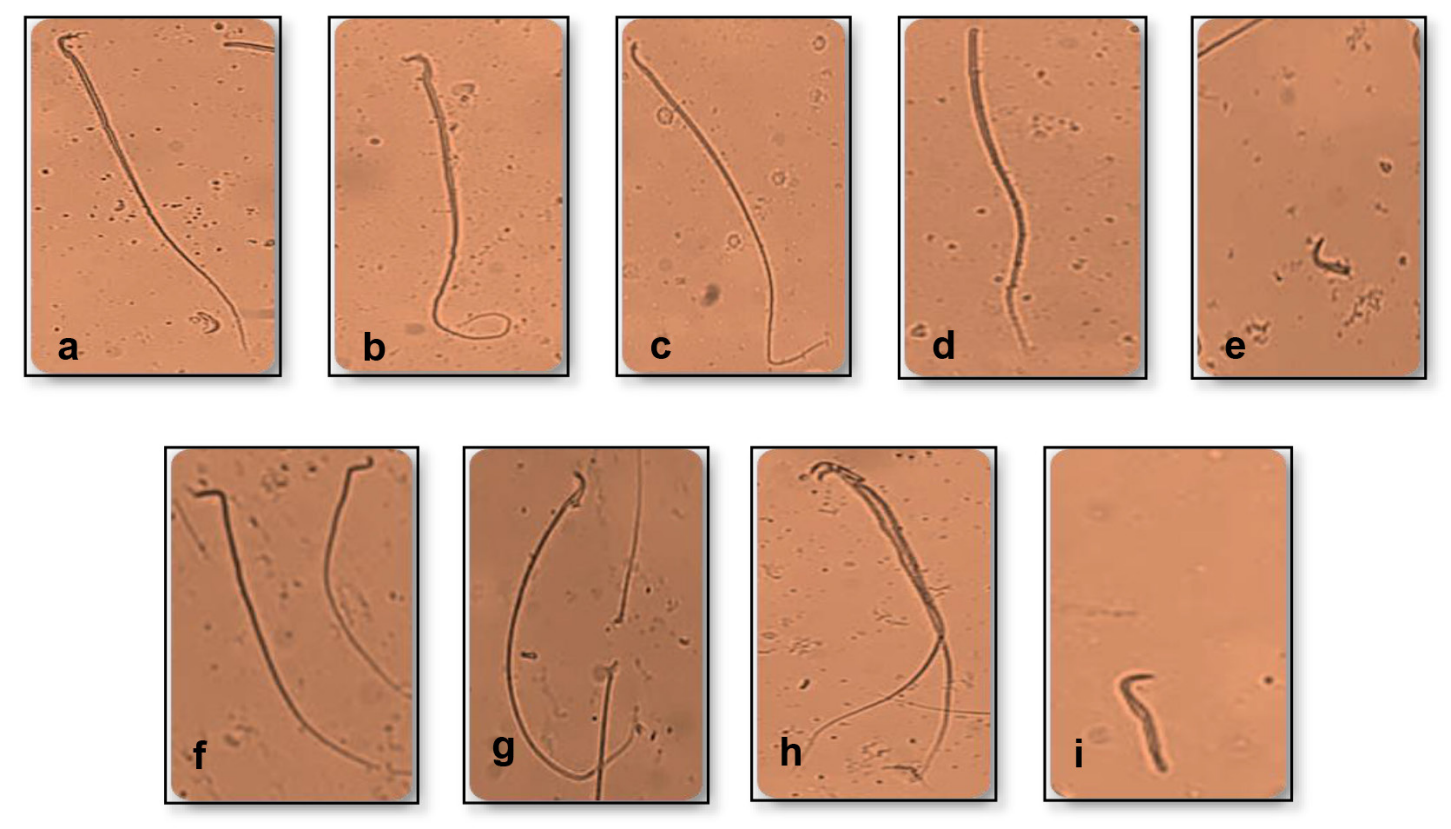
The Sprague Dawley rats’ sperm morphology (i.e. normal and abnormal sperm) was observed under 400× magnifications. (a) Normal head and tail; (b) Coiled tail; (c) Bent tail; (d) Headless; (e) Tail-less; (f) Hookless; (g) Banana-shaped; (h) Fused; (i) Short tail).

Data for the percentage of abnormal sperm was shown in Table 2, whilst the normal and abnormal shapes of sperm in the control and treatment groups were shown in Fig. 2. Significant differences (*p* < 0.05) were observed among groups when assessed using one-way ANOVA. The T1 group was seemed to have the lowest percentage of abnormal sperm compared to control and other treatment groups. However, the percentage of abnormal sperm increased when the dose was higher.

### Sperm Motility

**Figure 3:**
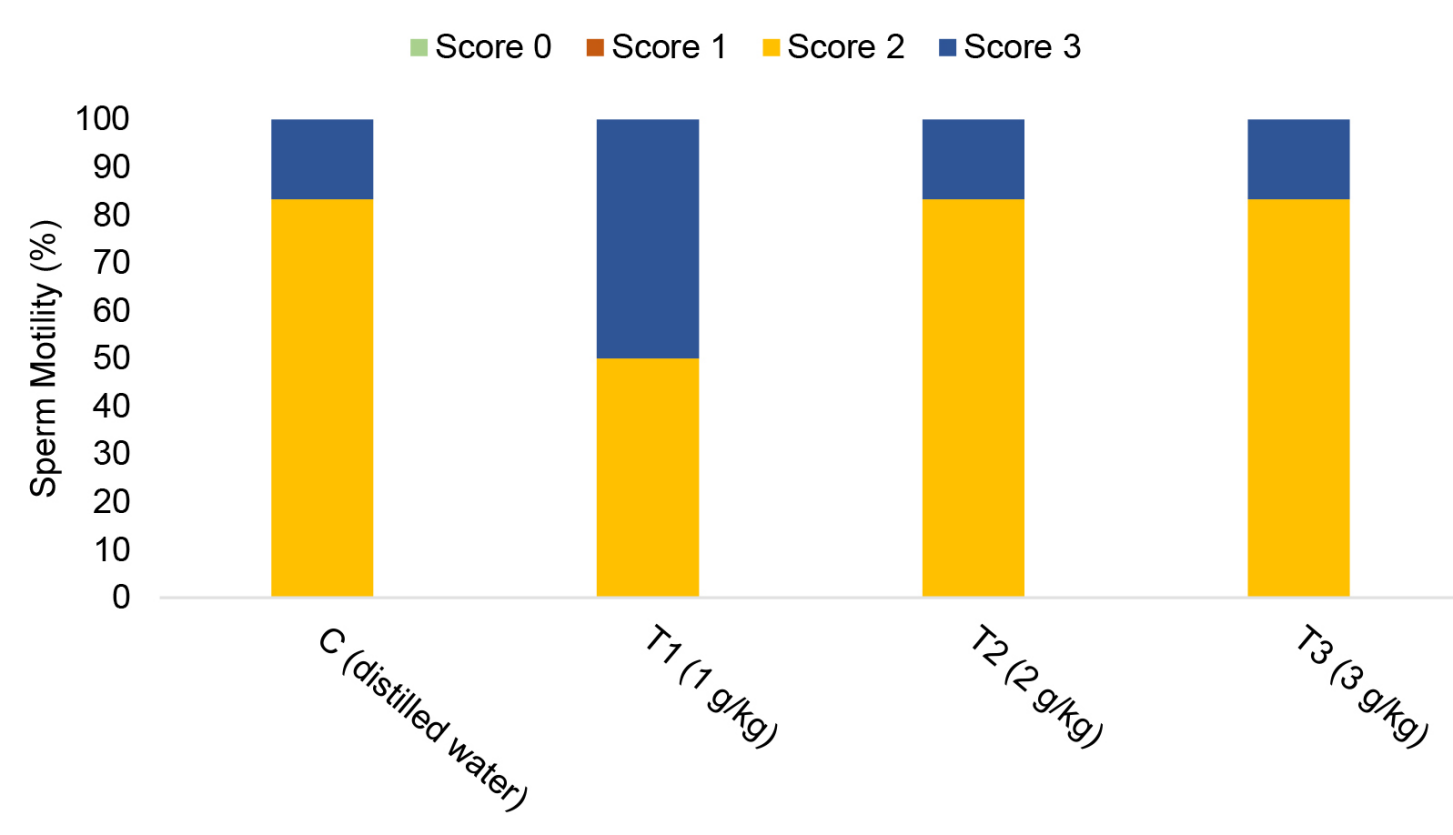
Sperm motility grading for different treatment levels of Aquilaria malaccensis in male Sprague Dawley rats (score 0 = not moving at all/immotile; score 1 = moving very slowly/moving with no forward progression; score 2 = moving slowly/moving with slow and wandering movement; score 3 = moving quickly/moving rapidly in an almost straight line. Data are presented as percentage (*n* = 6/group)).

Fig. 3 shows the results of sperm motility in control and treatment groups. The T1 group appeared to have the highest score of 3 (50%) compared to other groups. Majority of the group has received score 2 (80%), and no group obtained score 1 or 0. However, no significant differences were found among the groups (*p* > 0.05).

## DISCUSSION

Reproductive organ weight is an important index to indicate the weight changes for physiological and pathological status as the changes in reproductive organs weight could influence the function of that organs. For example, the changes in the testicular weights reflect changes in seminiferous tubules where its reduction or addition indicate a significant decline or increment in sperm production (Elangovan *et al.* 2006; Woldemeskel 2017). In this study, no significant differences were seen in reproductive organs weight between the treated and control groups. A recent study also has reported no changes in the weight of testis and epididymis among the groups when treated with *A. malaccensis* extract (100 mg/kg, 300 mg/kg and 500 mg/kg of rats’ body weight) (Razak *et al.* 2019a; 2019b). Zulkifle *et al.* (2018) also reported the same findings, where there was no abnormality found in testicular weight of treated rats (250 mg/kg and 500 mg/kg of rats’ body weight) compared to the control group. Although the dosage of *A. malaccensis* used in this study were higher than the previous studies, the results were still consistent. However, another study using different type of plant has showed a significant reduction in testis weight when using a higher dosage (3 g/kg of rats’ body weight) of *Vernonia amygdalina* leave treated in rats. Based on these findings, the different of weight changes in reproductive organs may vary depending on the species leaves used, the amount of dosage, duration of the treatment and the type of animal used. Thus, the supplementation of 1 g/kg, 2 g/kg and 3 g/kg of *A. malaccensis* extracts for 28 days did not likely affect reproductive organs’ weight.

As presented in Table 2, the oral administration of *A. malaccensis* leaves extract at 1 g/kg of rats’ body weight for 28 days has caused a significant increase (*p* < 0.05) in sperm concentration compared to control group. The high property of antioxidant such as flavonoids, alkaloids, tetripenoids, phenols, tannin, saponins and free radical scavenging activity in *A. malaccensis* leaves extract could enhance the number of sperm (Samsulrizal *et al.* 2011; Ismail *et al.* 2019). These bioactive compounds could probably protect spermatogonial stem cells against oxidative damage and improve the production of sperm. The extract also contains phenol group compounds, such as flavonoids, which are the main compounds that cause potent antioxidants. A phytochemistry study also revealed the presence of steroids in aqueous extract of *A. malaccensis* (Nik *et al.* 2014). Norraidah and Mahanem (2015) reported that the administration of steroid plants stimulates the reproductive organs related to sexual behaviour. A previous study conducted by Razak *et al.* (2019a; 2019b) has demonstrated that *A. malaccensis* showed a potential to ameliorate the toxicity induced by cyclophosphamide and improved sperm quality. Finding from this study also conforms to Ruyani and Sundaryono (2008) where the use of 1 g/kg body weight of *A. malaccensis* bark extract had improved sperm concentration and sperm motility in mice.

However, the present study observed a decrement in sperm concentration in T2 and T3 groups, respectively. Higher dosage at more than 1 g/kg of *A. malaccensis* leaves extract showed some adverse effects on sperm concentration as indicated in Table 2. The negative effects could be caused by some chemical constituents present as naturally safe but may exhibit toxic effects at a certain dose or prolonged exposure (Bode & Dong 2015). This result is consistent with the study by Adam *et al.* (2018) in which methanol extract of *A. malaccensis* has cytotoxicity and genotoxicity effects in lymphocytes at higher concentration. The process of spermatogenesis and the function of accessory reproductive organs are androgen-dependent (Desjardins 1978; Dym *et al.* 1979). Decreased androgen production reflected the decrease in mature Leydig cells and their functional status (Nusier *et al.* 2007). The chemical constituents in the extract may probably act on the pituitary gland and decrease the main hormone of spermatogenesis (Dym *et al.* 1979). Thus, the production of sperm was negatively affected. As mentioned before, there were a lot of research have been done using lower dosage of *A. malaccensis* (≤1 g/kg) in previous study. Since Zulkifle *et al.* (2018) has suggested the lethal dose (LD_50_) of *A. malaccensis* extract is above than 2 g/kg body weight, however higher dosages (2 g/kg and 3 g/kg body weight) were used in this study to investigate the effects of *A. malaccensis* in male reproduction. As this paper is the first to use a high dosage of *A. malaccensis* leaves extract in male reproductive system, the specific physiological mechanism for this reduction is still unclear. However, other potential factors may influence the production of sperm, such as a hormonal imbalance, since it plays an essential role in producing spermatogenesis, epididymal spermatozoa maturation, and sexual desire (Samsulrizal *et al.* 2011; Rasekh *et al.* 2015). Besides, testicular injury, epididymal toxicity or any spermatogenic disturbance will usually occur in decreased epididymal sperm count (Creasy & Chapin 2013). Therefore, further studies need to be undertaken to identify and isolate the active components in *A. malaccensis* leaves aqueous extract that affects fertility in male rats and determines its mechanism of action.

The significant decrease in percentage of abnormal sperm in T1 group could also be due to the high antioxidant property in the extract. It is well known that antioxidants play an essential role in protecting cells against free radical damage. According to Zakaria and Haron (2020), antioxidant components have a favourable influence on sperm morphology by reducing the number of abnormal sperm. This finding agrees with Razak *et al.* (2019a; 2019b), who also reported a similar discovery whereby the supplementation of *A. malaccensis* at dose 100 mg/kg for 63 days reduced the number of abnormal sperm. Although the dose used was slightly lower with longer treatment days, the effect on sperm could potentially be seen earlier and faster if the dose used is more effective and acceptable in body system. Moreover, the increment of the percentage of abnormal sperm in T2 and T3 groups might be due to excessive dosage use. According to Cock (2015), the prolonged consumption of high dosage might trigger adverse effects in sperm production as it may cause the sperm deoxyribonucleic acid (DNA) to denature and fragment, resulting in sperm abnormality (Gharagozloo & Aitken 2011). This observation is similar to Razak *et al.* (2019a; 2019b), where the administration of *A. malaccensis* at dose 2 g/kg body weight has shown adverse effects on the liver and kidney of rats. Thus, it is reasonable for the sperm to become abnormal when the dose was higher in both T2 and T3 groups. Khaki *et al.* (2009) also reported that exposure to toxic chemicals could lead to the production of abnormally shaped sperm. In addition, hormonal disturbance and disorders in spermatogenesis can also degrade the quality of sperm (Haron & Mohamed 2016). More studies are needed to identify active compounds that are responsible for sperm abnormality.

For the success of fertilisation, sperm must have the ability to move efficiently through the female reproductive tract. Poor sperm motility can be one of the factors of male infertility. Disturbance in testicular spermatogenesis or sperm flagellum function can affect the motility of the sperm. In rats, sperm motility is generally high and consistent between animals, but it varies between different laboratories (Creasy & Chapin 2013). The typical percentage of the total motility (Scores 1, 2 and 3) should be in the range of 85%–96% and the minimum acceptable value for sperm motility in control groups is 70% (Creasy & Chapin 2013). In the present study, although there were no significant differences among the groups, all groups appeared to have progressive sperm motility (Scores 2 and 3). In addition, this finding is consistent with a previous study that stated no significant difference in sperm motility among groups supplemented with *A. malaccensis* (Razak *et al.* 2019a; 2019b). Replication of the study with a higher sample size of animal and more extended treatment period are recommended to prove the validity of the findings.

## CONCLUSION

This research found that 1 g, 2 g and 3 g of *A. malaccensis* extract per kg body weight of male Sprague Dawley rats did not affect the weight of their reproductive organs. However, sperm count and sperm morphology were positively affected by the administration of *A. malaccensis* at 1 g per kg of rat’s body weight. Whilst, adverse effect was observed with higher dosages of *A. malaccensis* supplemented to the rats. This study suggests that 1 g of *A. malaccensis* extract per kg body weight of male Sprague Dawley rats is an effective dosage to be used to improve male fertility. However, further studies are needed to investigate and validate the present study’s findings with the use of bigger sample size, variation of treatment durations, and detail examination on physiological mechanism.

## Figures and Tables

**Table 1:** The effect of different levels of Aquilaria malaccensis leaves aqueous extract on reproductive organs’ weight (g).

Male reproductive organ	*n*	Control (distilled water)	Treatment 1 (1 g/kg)	Treatment 2 (2 g/kg)	Treatment 3 (3 g/kg)
Testis	*6*	1.46 ± 0.038	1.50 ± 0.034	1.44 ± 0.049	1.43 ± 0.045
Epididymis	*6*	0.40 ± 0.026	0.46 ± 0.009	0.45 ± 0.023	0.43 ± 0.014
Prostate gland	*6*	0.27 ± 0.020	0.26 ± 0.018	0.28 ± 0.030	0.27 ± 0.011
Seminal vesicle	*6*	0.24 ± 0.012	0.25 ± 0.011	0.24 ± 0.002	0.24 ± 0.008

*Notes*: Data are presented as mean ± SEM. No significant differences were found among the groups analysed by using one-way ANOVA test. The g/kg = gram of *Aquilaria malaccensis* extract per kilogram of rats’ body weight.

**Table 2:** The effect of *Aquilaria malaccensis* leaves aqueous extract on sperm concentration and sperm morphology.

Parameter	Treatment group			

	Control (distilled water)	Treatment 1 (1 g/kg)	Treatment 2 (2 g/kg)	Treatment 3 (3 g/kg)
Sperm concentration (×10^−6^)	1.06 ± 0.058^a^^b^	1.36 ± 0.030^b^^c^	1.01 ± 0.035^c^	0.78 ± 0.094^a^
Sperm morphology (%)	11.67 ± 0.333^a^^b^	8.17 ± 0.167^b^^a^	12.17 ± 0.307^a^^c^	18.83 ± 0.477^c^

*Notes*: Data are presented as mean ± SEM (*n* = 6/group). Significant differences were determined by one-way ANOVA followed by Tukey’s post hoc test with *p* < 0.05.

a *p* < 0.05 compared with the control group;

b *p* < 0.05 compared with T1 (1 g/kg) group;

c *p* < 0.05 compared with T2 (2 g/kg) group.
